# Mice Lacking beta2-Integrin Function Remain Glucose Tolerant in Spite of Insulin Resistance, Neutrophil Infiltration and Inflammation

**DOI:** 10.1371/journal.pone.0138872

**Published:** 2015-09-25

**Authors:** Paul J. Meakin, Vicky L. Morrison, Claire C. Sneddon, Terhi Savinko, Liisa Uotila, Susan M. Jalicy, Jennie L. Gabriel, Li Kang, Michael L. J. Ashford, Susanna C. Fagerholm

**Affiliations:** 1 Divison of Cardiovascular & Diabetes Medicine, School of Medicine, University of Dundee, Ninewells Hospital and Medical School, Dundee, United Kingdom; 2 Division of Cancer Research, School of Medicine, University of Dundee, Ninewells Hospital and Medical School, Dundee, United Kingdom; 3 Institute of Biotechnology, University of Helsinki, Helsinki, Finland; University of Minnesota - Twin Cities, UNITED STATES

## Abstract

Beta2-integrins are important in leukocyte trafficking and function, and are regulated through the binding of cytoplasmic proteins, such as kindlin-3, to their intracellular domain. Here, we investigate the involvement of beta2-integrins in the regulation of metabolic disease using mice where the kindlin-3 binding site in the beta2-integrin cytoplasmic tail has been mutated (TTT/AAA-beta2-integrin knock-in (KI) mice), leading to expressed but dysfunctional beta2-integrins and significant neutrophilia *in vivo*. Beta2-integrin KI mice fed on a high fat diet showed normal weight gain, and normal accumulation of macrophages and lymphocytes in white adipose tissue (WAT) and liver, but increased neutrophil numbers especially in WAT. In addition, beta2-integrin KI mice fed on a high fat diet showed significantly increased peripheral insulin resistance in response to high-fat feeding. However, this was associated with improved glucose disposal following glucose load. Interestingly, beta2-integrin KI neutrophils produced more elastase *in vitro*, in response to stimulation. Beta2-integrin KI mice displayed variability of tissue inflammatory status, with liver and WAT exhibiting little or no difference in inflammation compared to high fat fed controls, whereas skeletal muscle demonstrated a raised inflammatory profile in association with higher elastase levels and diminished signalling through the IRS1-PKB pathway. In conclusion, although expression of dysfunctional beta2-integrins increased neutrophil production and infiltration into tissue, skeletal muscle was the most affected tissue exhibiting evidence of higher neutrophil activity and insulin resistance. Thus, beta2-integrins modulate glucose homeostasis during high fat feeding predominantly through actions on skeletal muscle to affect metabolic phenotype *in vivo*.

## Introduction

Low-grade chronic inflammation and the increased expression of pro-inflammatory cytokines in adipose tissue, liver and skeletal muscle is associated with insulin resistance in obesity and type 2 diabetes. In addition to many other leukocyte populations, infiltration of neutrophils and production of the protease elastase by these cells has recently been implicated in the low-grade inflammation in adipose tissue and liver associated with metabolic disease [1, 2].

Beta2-integrins (CD18) are adhesion receptors found on the surface of leukocytes, and are major regulators of leukocyte trafficking and function. Interestingly, these receptors have been previously implicated in obesity and metabolic regulation [3, 4], although the molecular mechanisms involved remain poorly understood. Beta2-integrins are regulated through interactions with cytoplasmic regulators such as talin, kindlin-3, filamin and 14-3-3 proteins, factors which interact with the beta2-integrin cytoplasmic tail and regulate integrin function [5]. We have recently reported that mutation of the kindlin-3 binding site in the beta2-integrin intracellular domain results in expressed but dysfunctional integrins in T cells [6]. Interestingly, these mice also display increased neutrophil numbers in the bloodstream as well as in the bone marrow, and increased neutrophil survival *in vitro* [6, 7]. In addition, they display increased dendritic cell activation, cytokine production and dendritic cell-induced Th1 polarization, indicating that beta2-integrins play important roles in immune homeostasis *in vivo* [7]. Neutrophilia has also been described in beta2-integrin knockout mice and is a feature of leukocyte adhesion deficiency patients, where beta2-integrin expression or function is reduced or lost [8–11]. We therefore decided to investigate the role of beta2-integrins in the regulation of leukocyte trafficking, inflammation and metabolic phenotype in high-fat diet fed animals.

We report here that dysfunction of beta2-integrins does not affect trafficking of T cells or macrophages into adipose tissue or liver during high fat feeding, but numbers of neutrophils are significantly increased in the bloodstream and adipose tissue of these mice. In addition, although high fat fed beta2-integrin KI mice do not display increased levels of elastase in the liver or WAT or indeed increased inflammation in these tissues, there was an increase in elastase and inflammatory mediators in skeletal muscle, associated with systemic insulin resistance in these mice. Therefore, beta2-integrins do not promote leukocyte trafficking into tissues during high fat feeding, but instead *restrict* neutrophil production and tissue infiltration *in vivo*. In addition, beta2-integrins regulate the metabolic phenotype mainly through effects on skeletal muscle inflammation status *in vivo*. Therefore, beta2-integrin function plays important roles in restricting inflammation and metabolic disease during high fat feeding.

## Materials and Methods

### Mice and high fat feeding

The beta2^TTT/AAA^ integrin knock-in (beta2-integrin knock-in mice) mice have been previously described [6]. Mice were maintained in the Wellcome Trust Biocentre at the University of Dundee, in compliance with U.K. Home Office Animals (Scientific Procedures) Act 1986 guidelines (project licence number 60/4280). All animal care, experimental protocols and procedures were performed in accordance to the Animal Scientific Procedures Act (1986), with approval of University of Dundee Ethics Committee. Aged-matched male wild type (WT) and homozygote beta2^TTT/AAA^ integrin knock-in [6] mice were placed on a high fat (HF, 45% Kcal fat, TestDiets) diet, at 8–10 weeks of age, for 20 weeks.

### Glucose and insulin tolerance tests

After 20 weeks on the diet, glucose homeostasis was measured using glucose (GTT) and insulin (ITT) tolerance tests. GTT (2 mg/kg) and ITT (0.75 U/kg) tolerance tests were performed on mice starved overnight or for 4 hours, respectively. Blood glucose concentrations were measured for 2 hours post-injection using the Ascensia Breeze blood glucose monitoring system (Bayer Healthcare). To measure pancreatic β-cell function, glucose stimulated insulin secretion (GSIS) studies were performed. Mice were dosed with glucose (2 mg/kg) following an overnight fast, and blood samples were taken over 30 minutes for insulin measurements. Trunk blood was taken at the end of the study for measurement of insulin (Crystal Chem.) and leptin (R&D Systems) by ELISA according to manufacturers’ instructions.

### *In vivo* metabolic analysis

Mice were weighed weekly throughout the 20 week study. Fat and lean masses were measured using EchoMR 4in1-500 (EchoMRI). Food intake, oxygen consumption and RER were measured using Oxymax CLAMS system (Columbus Instruments). Mice were placed in the metabolic cages and allowed to habituate for 24 hours, and data collected and analysed from the subsequent 48 hours.

### Flow cytometry

At the end of the 20 week study, blood, perigenital white adipose tissue (WAT), liver and spleen were harvested from mice following overnight starvation for analysis by flow cytometry. Tissue samples were weighed, cut into small pieces using a scalpel, and digested in 1 mg/ml collagenase, type II (Life Technologies) plus 10 U/ml DNAse I (Sigma) for 30 minutes (spleen) or 1 hour (liver and WAT), with 20 mM EDTA (Sigma) added for the final 5 minutes of digestion. After digestion, samples were passed through a 100 μm filter. Blood samples were collected in EDTA (to a final concentration of 12.5 mM, Sigma), and 100 μl of blood taken for flow cytometry analysis after red blood cell lysis in Ack buffer (Life Technologies). Single-cell suspensions of lymphoid tissues were prepared. The following conjugated antibodies were used (from eBioscience unless otherwise stated, clones given in brackets): CD4 (RM4-5), CD8a (53–6.7), B220 (RA3-6B2), F4-80 (BM8), Gr-1 (RB6-8C5) CD11b (M1/70; BD Bioscience), CD11c (N418; Biolegend), CD25 (PC61), CD44 (IM7; BD Bioscience), CD69 (H1.2F3), CD206 (C068C2; Biolegend), CD86 (GL1; BD Bioscience). Fc block (clone 2.4G2; BD Bioscience) was included in all stains. DAPI (Life Technologies) was added to samples immediately before acquisition on an LSR Fortessa flow cytometer (Becton Dickinson). Data were analysed using FlowJo software (TreeStar). Cell numbers were calculated as % live cells and number of cells per g tissue, or number of cells per 100 μl blood.

### H&E staining and insulin staining

Pancreata were fixed in 4% paraformaldehyde in phosphate buffer solution (PBS) for 24 hours at 4°C, then paraffin embedded and cut into 5μm sections for immunohistochemistry or 3 μm for histology. Sections were cleared of wax and following an antigen retrieval step were permeabilised in PBST (PBS + 0.5% Triton X-100) for one hour then blocked in 5% BSA in PBST for one hour. Sections were then incubated with primary antibody in blocking solution (insulin 1:100 and glucagon 1:100; both Abcam), in a humidified chamber overnight at 4°C. After removal of primary antibodies, sections were incubated with appropriate secondary antibodies in PBST (Cy3 (Jackson ImmunoResearch) 1:250 and Alexa Fluor 488 (Life Technologies) 1:500), secondary antibodies removed and sections dehydrated and dried prior to coverslip placement with Vectashield anti-fade mounting medium (Vector Laboratories) and sealed with nail varnish. Slides were stored at 4°C until imaged. Images were acquired using a confocal laser scanning microscope (Leica TCS SP5 II). Islet size was estimated by measuring the diameter of the islet (ImageJ) from non-serial sections of pancreatic tissues.

### qPCR

At the end of the 20 week study, tissues were harvested following overnight starvation. RNA was extracted using Trizol (Sigma) and cDNA subsequently synthesised with Superscript II (invitrogen). Quantitative PCR was performed using assay-on-demand premixed TaqMan® primers and probes (Life Technologies), as follows: Arg-1 Mm00475988_m1; F4/80 Mm00802529_m1; IL-1β Mm00434228_m1; IL-6 Mm00446190_m1; NOS2 Mm00440488_m1; MCP-1 Mm00441242_m1; TNFα Mm00443260_g1; elastase Mm01168928_g1; NLRP3 Mm00840904_m1; caspase-1 Mm00438023_m1; actin 4352933E.

### Immunoblotting

After an overnight fast tissues (quadriceps muscle, liver and perigenital fat) were collected in liquid nitrogen. Tissue samples were ground with a pestle and mortar under liquid nitrogen and homogenized in a protein lysis buffer. Samples were spun at 13,000rpm for 15 minutes and the protein content of the supernatant was determined using the Bradford method. Proteins (20μg) were separated by SDS-PAGE and transferred to nitrocellulose membranes and membranes incubated for 1 hour with fluorescently tagged secondary antibodies (Li-Cor Biosciences) and visualised using the odyssey infrared imaging system (Li-Cor Biosciences). Equal protein loading of samples was assessed by actin. GLUT4 expression was performed on samples that were not boiled to allow detection of membrane proteins. Antibodies against protein kinase B (PKB), phosphorylated PKB (p-PKB), insulin receptor substrate-1 (IRS-1), phosphorylated IRS-1 (p-IRS-1) and tumour necrosis factor alpha (TNFα) were purchased from Cell Signaling (Invitrogen). The antibodies against glucose transporter 4 (GLUT4), Elastase, interleukin-1 beta (IL-1β), interleukin-6 (IL-6) were obtained from Abcam and actin from Sigma. All antibodies used at 1:1000 dilution.

### Elastase activity assay

Neutrophils were purified from WT and KI bone marrow by negative selection using a Neutrophil Isolation Kit (Miltenyi Biotec) according to the manufacturer’s instructions. Purified neutrophils were suspended in 1% BSA/RPMI 1640, seeded onto a 96-well plate (1 x 10^6^ cells/well), and activated with lipopolysaccharide (LPS) or phorbol 12-myristate 13-acetate (PMA) in the presence of granulocyte-macrophage colony-stimulating factor (GM-CSF) for 4 h. Elastase activity was detected from cell supernatants with a Neutrophil Elastase Activity Assay Kit (Cayman Chemicals), according to the manufacturer’s instructions.

### Statistics

All data are presented as mean +/- SEM. The two-tailed Student’s *t*-test, ANCOVA (analysis of co-variance) or ANOVA, with a Bonferroni post hoc analysis, as appropriate (Graphpad Prism) were used to calculate statistical significance. *p<0.05, **p<0.01, ***p<0.001 versus control.

## Results

### Normal weight gain in beta2-integrin KI mice fed a high fat diet

**Fig 1:**
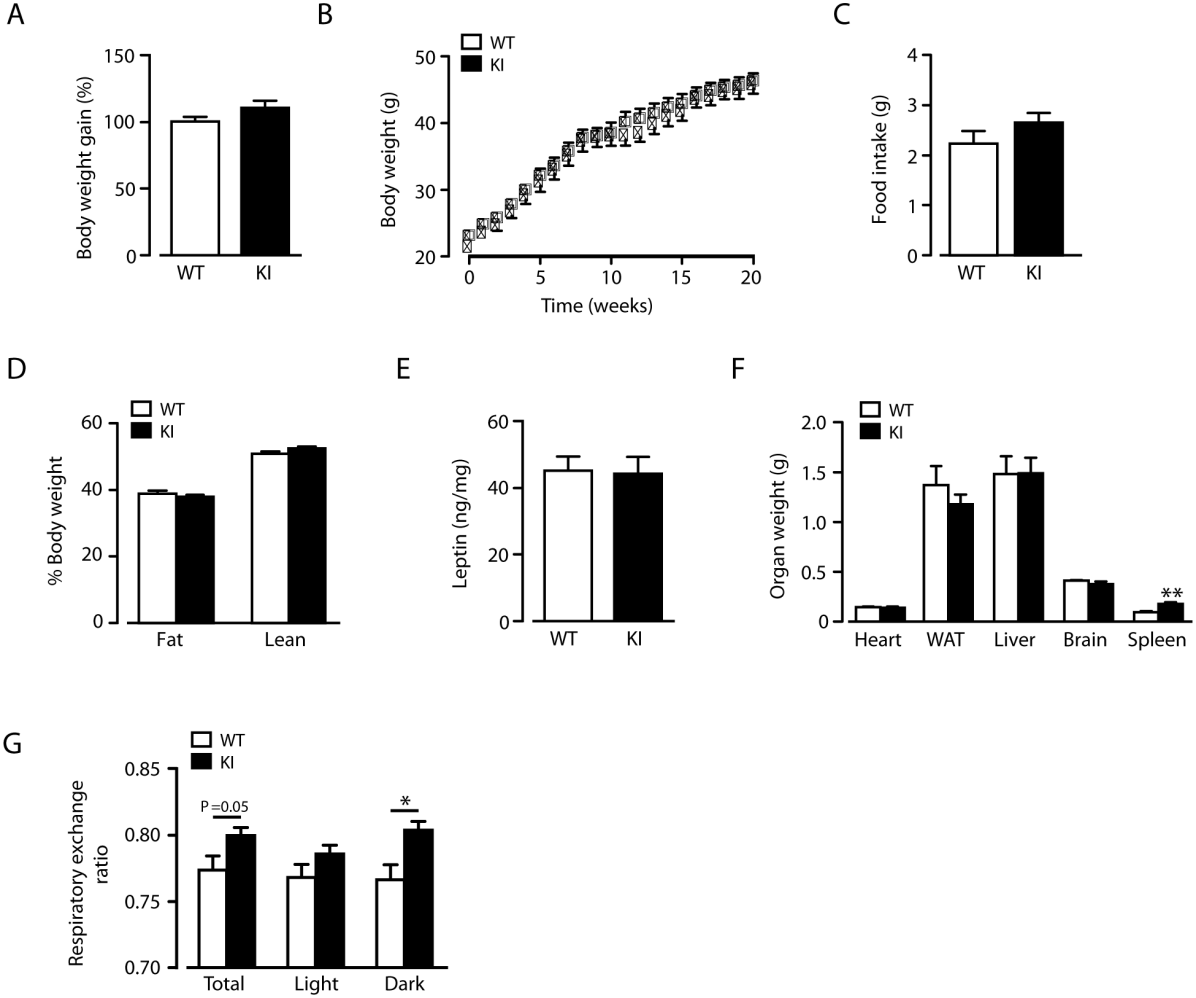
Normal weight-gain of beta2-integrin knock-in mice on a high fat diet. (A) Weight gain by WT and KI mice on a HF diet. (B) Body mass curves of age-matched WT and KI mice fed on a HF diet, monitored over a period of 20 weeks from 8–10 weeks of age. (C) Mean food intake per day of HF-fed WT and KI mice (D) Percentage lean and fat mass determined by qMR imaging in HF-fed 7-month-old male WT and KI mice. (E) Fasting blood leptin levels after 20 weeks HF-feeding for WT and KI mice. (F) Weights of tissues from WT and KI mice at the end of the 20-week HF-feeding study. (G) Respiratory exchange ratio (RER) for WT and KI mice at 20 weeks of HF diet; denoting overall mean RER over the 48 hour period and during light and dark phases. (*n* = 8–11. The results are means ± S.E.M. * *P* < 0.05; ** *P* < 0.01.

Leukocyte infiltration and low-grade inflammation is an important component of metabolic dysfunction during high fat feeding, and beta2-integrins are important for leukocyte trafficking but also restrict inflammation in various settings *in vivo*. We have previously reported that disrupting the link between the beta2-integrin and its cytoplasmic regulator, kindlin-3, results in expressed but dysfunctional integrins, leading to reduced T cell trafficking, but also to neutrophilia and increased dendritic cell activation and Th1 polarization *in vivo* [6, 7]. We therefore decided to investigate the response of TTT/AAA-beta2-integrin KI mice, where the integrin/kindlin link is disrupted and the integrins are expressed but dysfunctional, [6, 7], to challenge with a high fat (HF) diet. Weight gain in KI mice was indistinguishable to that of wild type littermates when fed a HF (45%) diet (Fig 1A) for 20 weeks, resulting in both genotypes achieving the same level of obesity (Fig 1B). There was no difference in mean daily food intake (Fig 1C) during HF feeding and both genotypes exhibited identical expanded fat mass (Fig 1D) and increased plasma leptin levels (Fig 1E), consistent with an equivalent obese phenotype. Furthermore, adipose tissue weights were unaltered in comparison to HF-fed wild type mice in HF-fed beta2-integrin KI mice, as were weights of all other organs except the spleen (Fig 1F), which has increased cellularity due to altered leukocyte migration patterns [6]. An unaltered fat distribution has previously been reported in CD11c knockout mice [12]. Energy expenditure, as determined by O_2_ consumption, was unaffected by the beta2-integrin mutation (data not shown). However HF-fed beta2-integrin KI mice displayed an overall higher respiratory exchange ratio compared to HF-fed wild type mice, shown as a significant increase in the dark phase (Fig 1G), indicating increased carbohydrate oxidation, also observed in fasted CD18-deficient mice [3]. Therefore, dysfunction of beta2-integrins does not impact on overall body fat deposition in mice challenged with HF diet.

### High-fat fed beta2-integrin knock-in mice display increased insulin resistance and improved glucose disposal

**Fig 2:**
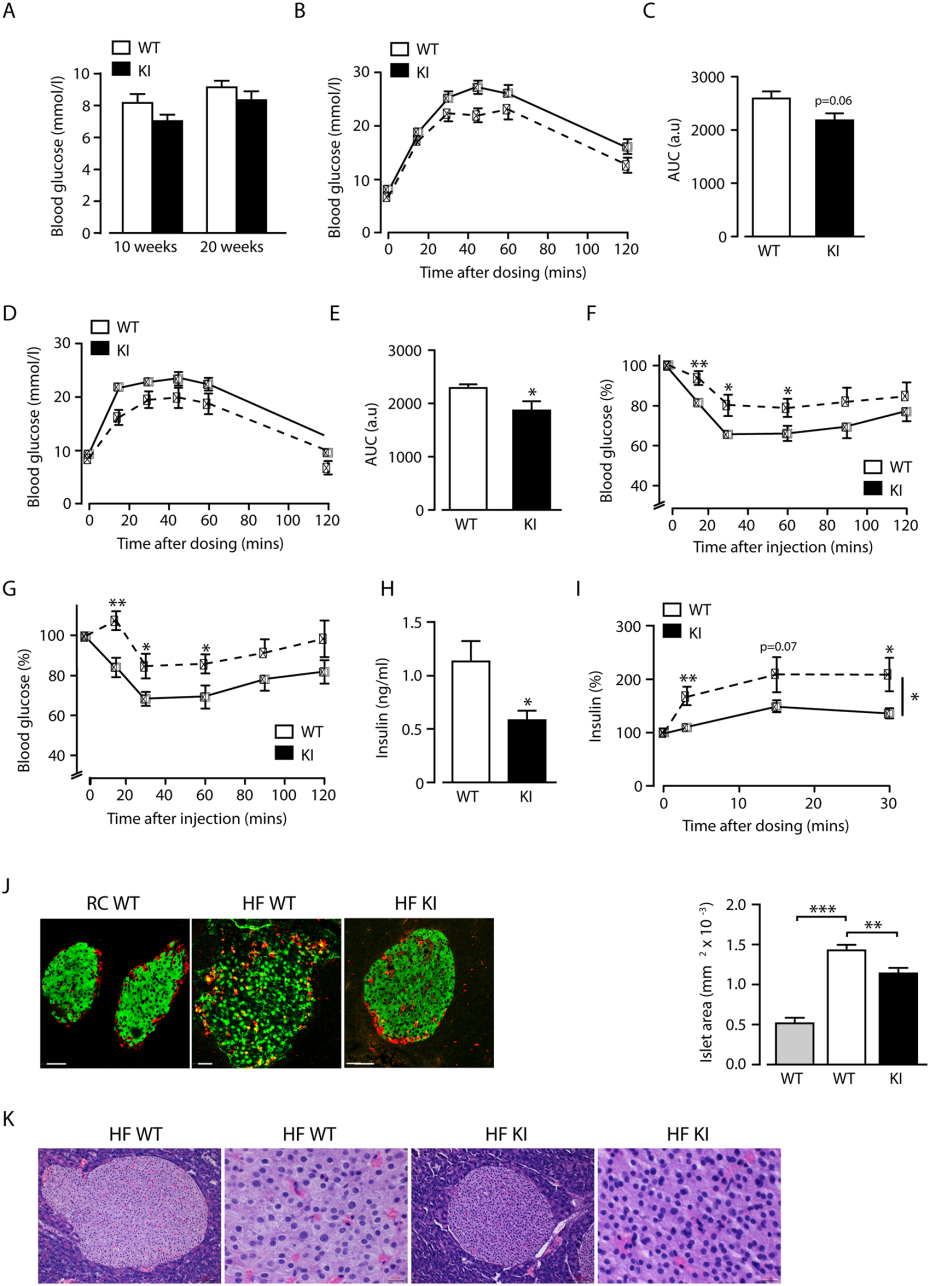
Reduced insulin sensitivity with increased glucose clearance by beta2-integrin knock-in mice fed a high fat diet. (A) Fasted blood glucose levels after 10 and 20 weeks HF-feeding in mice of the indicted genotypes (B-E) Intraperitoneal glucose tolerance tests were performed on WT and KI mice after 10 (B) and 20 weeks (D) of HF-feeding, with quantification of the total glycaemic excursion (area under the curve; AUC) shown at 10 (C) and 20 (E) weeks. Insulin tolerance tests were performed on WT and KI mice after 10 (F) and 20 (G) weeks of HF-feeding. (H) Fasted blood insulin levels after 20 weeks HF-feeding for WT and KI mice. (I) Glucose stimulated insulin secretion in WT and KI mice after 20 weeks of HF-feeding. *n* = 8–11. (J) (left panel) Representative confocal images of pancreatic islets from RC- and HF-fed WT and HF-fed KI mice, stained for insulin (green) and glucagon (red). Scale bar is 50 microns. (right panel) Islet areas for RC-fed WT mice (grey bar; n = 20; N = 2); HF-fed WT mice (white bar: n = 25, N = 4) and HF-fed KI mice (black bar). n = 39; N = 4). (K) Representative haematoxylin and eosin staining of pancreas showing no neutrophil infiltration in HF-fed WT and KI mice. Scale bar = 100 μm (in 10x magnification) and 20μm (in 40x magnification). The results are means ± S.E.M. * *P* < 0.05; ** *P* < 0.01; *** *P* < 0.001.

Neutrophils have been shown to play a role in insulin sensitivity of mice on a high fat diet [1]. Therefore, we went on to investigate glucose homeostasis during HF feeding in beta2-integrin KI mice, which displayed increased neutrophil numbers [6, 7]. In control experiments, on regular chow (RC) diet, we found no difference in glucose homeostasis or insulin sensitivity between wild type and beta2-integrin KI mice (S1 Fig). Fasted blood glucose levels of HF-fed beta2-integrin KI mice were not significantly different from HF-fed wild type controls (Fig 2A) with fasted glucose higher in both genotypes compared to RC fed mice (S1 Fig). Interestingly, the beta2-integrin KI mice displayed mildly improved glucose tolerance at both 10 and 20 weeks of HF feeding compared to wild type mice (Fig 2B–2E). In contrast, at both time points, beta2-integrin KI mice displayed an impaired glucose disposal response to insulin injection compared to wild type mice (Fig 2F and 2G). Further investigation revealed fasted insulin levels in KI mice to be significantly lower than wild type levels (Fig 2H), and KI mice responded to glucose injection from this basal level with an increased glucose-stimulated insulin secretion unlike wild type mice, which showed no significant increase in insulin levels from the fasted baseline (Fig 2I), although this was still attenuated compared to RC-fed wild type mice. Together, these data demonstrate that although HF-fed beta2- integrin KI mice are insulin resistant they have improved glucose disposal, possibly owing to increased basal (non insulin-dependent) glucose uptake associated with a higher rate of glucose oxidation and better residual beta cell function. The improved glucose tolerance and glucose-stimulated insulin secretion of the beta2-integrin KI mice led us to investigate whether there were differences in pancreatic morphology of these HF-fed mice. As expected HF-feeding increased islet area, indicative of expanded pancreatic beta cell mass, with alpha cell infiltration of islets in wild type mice (Fig 2J). However, islets were significantly smaller in HF-fed beta2-integrin knock-in mice, although alpha cell infiltration was apparent (Fig 2J). There was no evident infiltration of neutrophils in this organ (Fig 2K).

### Normal macrophage accumulation, but increased neutrophil accumulation in adipose tissue of high fat diet-fed beta2-integrin knock-in mice

**Fig 3:**
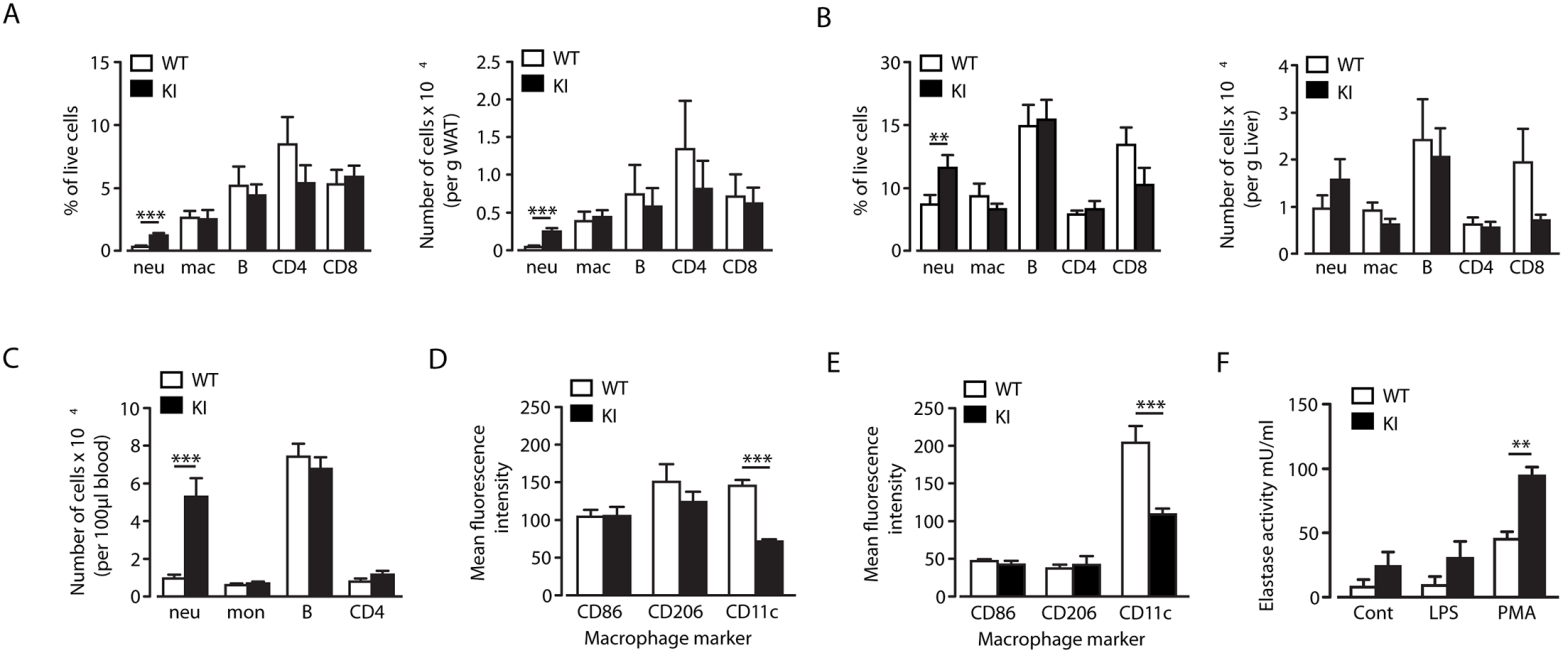
Increased neutrophil accumulation in adipose tissue of beta2-integrin knock-in mice, but normal macrophage infiltration and differentiation. (A-B) At the end of the 20-week HF-feeding study, WAT (A) and liver (B) from WT and KI mice were analyzed by flow cytometry to quantify numbers of leukocytes, shown as percentage of live cells (left) and number of cells per g tissue (right). (C) Blood samples were analysed by flow cytometry for leukocyte numbers, shown per 100μl blood. (D-E) F4/80+ macrophages in the adipose tissue (D) and liver (E) were analysed for expression of the M1/M2 macrophage markers, CD86, CD206 and CD11c. *n* = 10–11. (F) Elastase activity of isolated neutrophils from WT and KI mice, stimulated with LPS or PMA. *n* = 3. The results are means ± S.E.M. ** *P* < 0.01; *** *P* < 0.001.

Alpha4-integrins have been reported to play a role in macrophage accumulation in the adipose tissue during HF diet-induced obesity [13]. However, although the TTT-region of the beta2-integrin cytoplasmic domain regulates integrin-mediated adhesion under shear flow conditions and adhesion strength to ligand [6], macrophage accumulation in both white adipose tissue (WAT) and liver after 20 weeks of high fat feeding was normal in beta2-integrin KI mice (Fig 3A and 3B). In addition, the distribution of other immune cell types in these tissues was also relatively normal (Fig 3A and 3B). The exception to this was a higher number of neutrophils in WAT (Fig 3A), likely reflecting the elevated numbers of neutrophils in these mice (Fig 3C). Neutrophils were also increased in the liver, although the increase was not statistically significant (Fig 3B). In addition, there was a slight (non-significant) reduction in CD4 T cell numbers in WAT and of CD8 T cells in the liver (Fig 3A and 3B). Therefore, the TTT/AAA mutation in the beta2-integrin tail, which affects binding of leukocytes to integrin ligands under shear flow conditions [6] does not significantly affect trafficking of leukocytes, other than neutrophils, into WAT or liver under conditions of high fat feeding. These results are in agreement with previous results using ICAM-1- and CD11b-deficient mice [14].

To investigate inflammation status in HF-fed beta2-integrin KI mice, we analysed expression levels of CD86, CD206, and CD11c, the typical M1/M2 macrophage identification markers, in F4/80+ cells that had infiltrated the WAT and liver. CD86 and CD206 expression levels in WAT and liver of beta2-integrin KI mice were normal, whilst CD11c (which pairs with the beta2/CD18-integrin chain to form CD11c/CD18) expression was reduced (Fig 3D and 3E). As the TTT/AAA-mutation in the beta2-integrin tail affects integrin surface expression, the reduced CD11c expression is likely not a reflection of reduced M1-polarization, but a consequence of reduced integrin surface expression due to the mutation [6]. Therefore, there were no significant differences in macrophage polarization in WAT and liver of these mice.

### Tissue-specific increase in elastase expression and inflammation in beta2-integrin KI mice on a high fat diet

**Fig 4:**
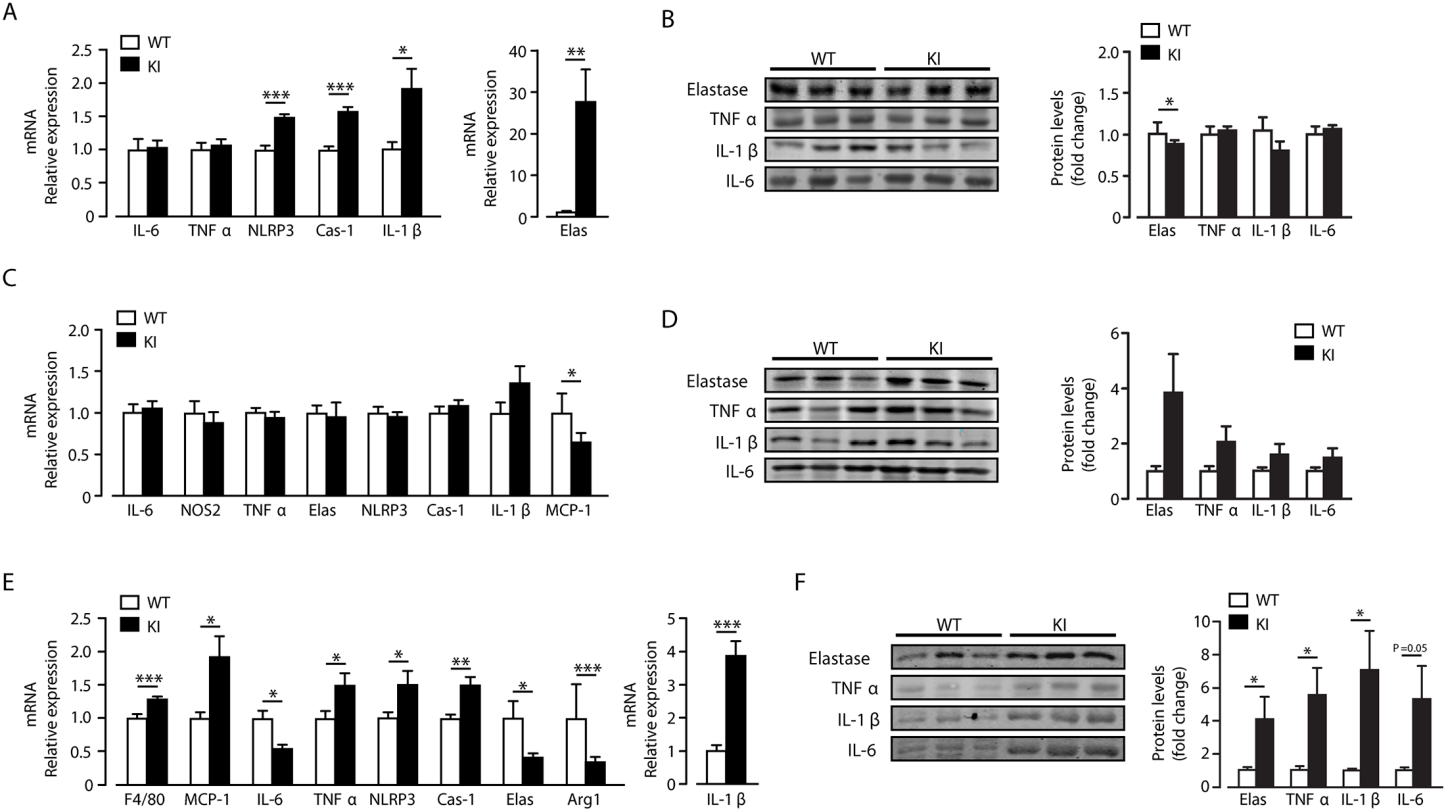
Elevated inflammation and elastase levels in skeletal muscle but not liver and WAT. After 20 weeks of HF feeding, gene expression levels of the pro-inflammatory markers; TNFα, IL-6, elastase, NLRP3, Caspase-1, and IL-1β were measured in the liver (A) WAT (C) and muscle (E) of WT and KI mice. Additionally, levels of NOS2 and MCP-1 are shown for WAT (D) and F4/80, MCP-1, Arg-1 for skeletal muscle (E) *n* = 5–11. Representative immunoblots for elastase, TNFα, IL-1β and Il-6 in liver (B), WAT (D) and skeletal muscle (F) with quantification of the immunoblot data for each protein also shown. *n* = 8–11. The results are means ± S.E.M. * *P* < 0.05; ** *P* < 0.01; *** *P* < 0.001.

Obesity is associated with increased circulating neutrophils in humans and rodents, with evidence to suggest that these are chronically activated [15–17]. Indeed, elastase production by neutrophils has recently been demonstrated to play a crucial role in inflammation and obesity in response to high fat diet feeding [1, 2]. Interestingly, in addition to increased neutrophil numbers in blood, WAT and liver of beta2-integrin KI mice, *in vitro* analysis of isolated neutrophils demonstrated that the TTT/AAA integrin mutation resulted in enhanced elastase production in response to phorbol ester stimulation compared to wild-type cells (Fig 3F). This result, in conjunction with the creased insulin resistance associated with the beta2-integrin KI mice, led us to examine elastase levels and WAT, liver and muscle inflammatory status in more detail. Surprisingly, there was no evidence to indicate that the enhanced trafficking of neutrophils into liver had any significant impact on inflammatory status. Thus although mRNA expression of inflammasome components such as NLRP3 and caspase-1, along with IL-1β and elastase were increased in beta2-integrin KI, compared to wild type, livers, there was no increase detected in protein levels of TNFα, IL-1β and IL-6 and surprisingly a reduction in elastase levels (Fig 4A and 4B). There was also no clear indication of a modified pro-inflammatory response in beta2-integrin KI WAT versus controls, with mRNA expression of IL-6, NOS2, TNFα, NLRP3, caspase-1, IL-1β and elastase unchanged, with reduced MCP-1 expression (Fig 4C). Furthermore, levels of elastase, TNFα, IL-1β and IL-6 were unaltered in beta2-integrin KI WAT, although there was a trend towards increased levels for all of these proteins (Fig 4D). These data suggest that M1 macrophage differentiation and neutrophil activity in WAT and liver of beta2-integrin KI mice, and thus their inflammatory status, is largely unaltered by the TTT/AAA integrin mutation.

As skeletal muscle is the main tissue for glucose uptake in the body, we also examined the effect of the beta2-integrin mutation on its inflammatory status. In contrast to liver and WAT, skeletal muscle of beta2-integrin KI mice exhibited an increased pro-inflammatory status compared to controls. At transcript level, TNFα, IL-1β, NLRP3, MCP-1 and caspase-1 were increased, as was the expression of F4/80 (indicative of increased macrophage numbers) with decreases in IL-6 and arginase 1 (Fig 4E). Furthermore, protein levels of TNFα, IL-1β and IL-6 were also increased in beta2-integrin KI skeletal muscle (Fig 4F). Interestingly, elastase protein levels were raised in skeletal muscle of KI mice, opposite to the direction of mRNA change. A divergent transcript and protein expression outcome for elastase was also observed for beta2-integrin liver, albeit to a lesser extent.

**Fig 5:**
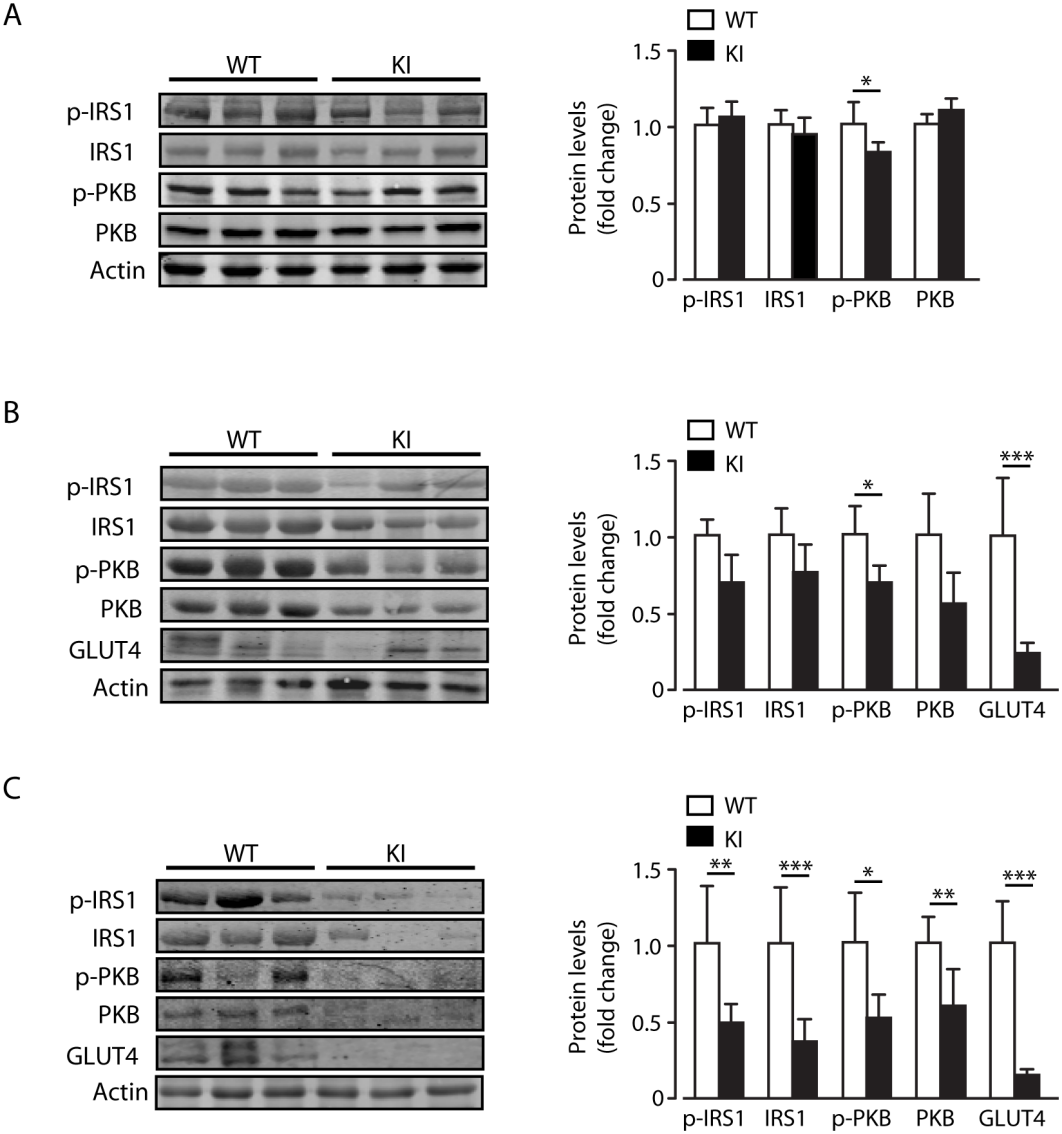
Increased inflammation in skeletal muscle of KI mice is associated with reduced basal IRS1-PKB signalling. (A-C) Representative immunoblots for p-IRS1, IRS1, p-PKB and PKB, with quantification of immunoblot data for each protein from liver (A), WAT (B) and skeletal muscle (C) of WT and KI mice. Immunoblots and protein levels of GLUT4 in WAT (B) and skeletal muscle (C) are also shown for WT and KI mice. *n* = 8–11. The results are means ± S.E.M. * *P* < 0.05; ** *P* < 0.01; *** *P* < 0.001.

Neutrophil elastase knockout mice have been described to have lower inflammatory tone than wild type mice on a HF diet, leading for example to reduced IL-1β expression [1]. Thus our findings that beta2-integrin KI mice on a HF diet, which have increased neutrophil infiltration and elastase levels and raised inflammatory tone in skeletal muscle, is consistent with an important role for neutrophil elastase in the inflammatory process. The expression of dysfunctional beta2-integrins in the beta2-integrin KI mice appears to have resulted in increased neutrophil production, neutrophil activation (as denoted by raised elastase levels) and inflammation in skeletal muscle, which may link with cellular insulin resistance [1]. Indeed, we find that skeletal muscle is the principal tissue affected when insulin signalling was examined. Thus, there was a significant decrease in basal IRS1, p-IRS1, PKB and p-PKB in KI muscle and p-PKB in KI WAT, which is not observed in KI liver, compared to control tissues (Fig 5A and 5C). We also detected a large decrease in GLUT4 protein levels in skeletal muscle and WAT (Fig 5B and 5C).

Taken together, these data indicate that macrophage recruitment to adipose tissue and liver during high fat feeding is normal in beta2-integrin knock-in mice and that the increased neutrophil infiltration has little impact on elastase levels and the overall inflammatory status or insulin sensitivity of these tissues. In contrast, skeletal muscle displays indicators of increased recruitment of macrophages and neutrophils leading to higher elastase levels and a pro-inflammatory state, consistent with a deleterious outcome on insulin sensitivity and the metabolic phenotype.

## Discussion

Excess nutrient intake drives expansion of adipocytes and increases secretion of chemoattractants (e.g. MCP-1 and leptin) and of cytokines (e.g. TNFα and IL1β) from WAT, which act to attract immune cells promoting dysfunctional lipid metabolism and further pro-inflammatory cytokine production and release. Generally obesity, in association with chronic inflammation in WAT and liver is closely associated with systemic insulin resistance [18, 19]. Furthermore, this obesity-driven combination of low insulin sensitivity with an inability of pancreatic beta cells to increase insulin secretion sufficiently to maintain glucose homeostasis, leads to hyperglycaemia and eventually to diabetes. Although the role of tissue macrophages, predominantly M1-like, has been the main focus of research into the connection between excess nutrient intake, low-grade chronic inflammation and insulin resistance, recent studies show significant contributions from other immune cell types (e.g. T-cells, B-cells and eosinophils). Neutrophils have also recently been shown to infiltrate adipose tissue and liver during high fat feeding [1]. Neutrophils secrete numerous proteases including elastase, which mediates many of the effects of neutrophils on adipose tissue and liver during high fat feeding, including insulin resistance [1]. Additionally neutrophils are a major site for expression of NLRP3 inflammasome components and a significant source of IL1β production [20, 21], processes demonstrated to be important in obesity-induced insulin resistance [22].

Neutrophilia is a significant symptom in murine models with defects in adhesion molecules, including a recently developed beta2^-^integrin knock-in (KI) mouse where the mutation abolishes the integrin-kindlin interaction [6–10, 23]. We show here that beta2-integrin regulates glucose metabolism during high fat feeding, as knock-in mice (which have a deficiency in beta2-integrin function due to lack of binding of the important cytoplasmic regulator kindlin-3) on a high fat diet display both insulin resistance but also improved glucose tolerance. We show that neutrophil infiltration into tissues, and elastase production predominantly in skeletal muscle is increased in mice with deficiency in beta2-integrin function. In addition, these mice display increased inflammation in skeletal muscle as demonstrated by higher levels of IL-1β, caspase-1 and NLRP3, probably explaining the increased insulin resistance in these mice. Indeed we find that insulin signalling efficacy is reduced, particularly in skeletal muscle of the KI mice. This is in agreement with previous findings that neutrophil elastase KO mice have reduced levels of IL-1β in the liver and serum [1] and that diminished NLRP3 inflammasome and caspase-1 activity elicits protection from high-fat diet mediated metabolic disorder [22].

Beta2-integrins are necessary for neutrophil trafficking into sites of inflammation. Therefore, it may appear contradictory that the beta2-integrin TTT/AAA mutation leads to increased, rather than decreased, levels of neutrophils in adipose tissue. However, neutrophil trafficking into inflammatory sites is not always dependent on beta2-integrins [14, 24], or the beta2-integrin/kindlin-3 interaction [25], and we show here that trafficking of leukocytes into adipose tissue and liver during high fat feeding is not dependent on the beta2-integrin/kindlin interaction. Rather, the increased numbers of neutrophils in the bone marrow and bloodstream of these mice appears to lead to higher numbers of neutrophils in tissues. Interestingly, isolated beta2-integrin KI neutrophils also produce more elastase in response to cell activation *in vitro*. *In vivo*, the increase in elastase levels in conjunction with increased NLRP3, caspase-1 and IL1β expression in skeletal muscle indicates increased neutrophil activity in this tissue in the beta2-integrin knock-in mice. Increased beta2-integrin KI neutrophil activation *in vitro*, and *in vivo* in response to high fat feeding, may be related to the increased responsiveness of beta2-integrin KI neutrophils to the growth factor GM-CSF that we have recently reported [7].

In contrast, the higher number of neutrophils infiltrated into WAT of beta2-integrin KI mice is not associated with significantly increased levels of elastase or inflammasome activity, compared to wild type mouse WAT, although there is a trend towards higher levels of these proteins. Furthermore, there is no evidence for increased numbers of macrophages or for a significant increase in pro-inflammatory cytokine levels in WAT of beta2-integrin knock-in mice on high fat diet, indicating there is not an exacerbation of the high fat diet mediated inflammatory response through either leukocyte population in this tissue. Liver tissue of beta2-integrin knock-in mice also demonstrated no increase in macrophage-dependent inflammation, with unchanged mRNA and protein levels of TNFα and IL-6 expression and macrophage numbers, which is consistent with the unchanged fasted blood glucose levels between beta2-integrin KI and control mice. Although neutrophil numbers in liver of beta2-integrin knock-in mice were not significantly raised we observed increased expression of inflammasome components and a large increase in elastase mRNA. However no change in protein levels of inflammatory cytokines could be detected and elastase levels were decreased. The dichotomy observed between elastase mRNA and protein levels in both liver and muscle suggests the presence of a negative feedback loop connecting tissue elastase activity and transcription of the elastase gene. Indeed, such a negative feedback mechanism linking neutrophil elastase activity to transcriptional repression of the elastase gene has been described in a myeloid leukemia cell line [26].

Although we have not been able to determine the distribution of immune cells directly in skeletal muscle following high fat diet, it is likely that there is an increase in macrophage numbers and (M1-like) activity associated with beta2-integrin knock-in, compared to wild type, mice. This is suggested by the higher expression of the macrophage marker F4/80 and the monocyte chemoattractant MCP-1. The increased expression of NLRP3, caspase-1 and IL-1β in KI, compared to wild type, skeletal muscle also indicate increased inflammation in this organ, which may be related to neutrophils as elastase levels in skeletal muscle were also increased. The increased expression of TNFα and IL-1β likely contribute to the insulin resistance observed in the KI mice, as increased expression of these cytokines in skeletal muscle are associated with higher insulin resistance [27, 28]. However, increased levels of elastase have also been reported to reduce IRS1-PKB protein levels and signalling [1]. Exactly how dysfunction of beta2-integrin leads to increased muscle inflammation is unknown at present and requires further studies.

In summary, the beta2-integrin mutation is associated with neutrophilia and under high fat diet conditions results in an obese phenotype, similar to that observed for wild type mice. For the same level of obesity, the beta2-integrin mutation alters local tissue inflammation, with predominantly skeletal muscle exhibiting increased elastase levels and inflammatory tone. The resultant altered expression of pro-inflammatory cytokines in skeletal muscle, may explain the systemic insulin resistance observed in HF-fed KI mice. The overall improved glucose tolerance of beta2-integrin mutation mice in response to raised blood glucose is most likely owing to higher basal (non-insulin dependent) glucose uptake, driven in part by higher glucose utilisation and better glucose-stimulated insulin secretion, which is possibly associated with reduced HF diet-mediated islet expansion. Further experiments such as hyperinsulinemic-euglycemic clamp studies, are now required to extend these observations and to examine the mechanisms underlying the selective influence this beta2-integrin mutation, under high fat diet, has on neutrophil interactions with skeletal muscle and skeletal muscle inflammation.

## Supporting Information

S1 Fig Normal glucose homeostasis of beta2-integrin KI mice on a regular chow diet. (A) Fasted blood glucose levels RC-fed mice of the indicted genotypes. (B) Intraperitoneal glucose tolerance tests were performed on RC-fed WT and KI mice, with quantification of the total glycaemic excursion (area under the curve; AUC) shown. (D) Fasted blood insulin levels for RC-fed WT and KI mice. (E) Insulin tolerance tests performed on RC-fed WT and KI mice. *n* = 4–8.
(TIF)
