# Supplementary figures and images for: Mice Lacking beta2-Integrin Function Remain Glucose Tolerant in Spite of Insulin Resistance, Neutrophil Infiltration and Inflammation
Source: PLoS One. 2015 Sep 25;10(9):e0138872. doi: 10.1371/journal.pone.0138872 (PMC4583187; doi:10.1371/journal.pone.0138872)

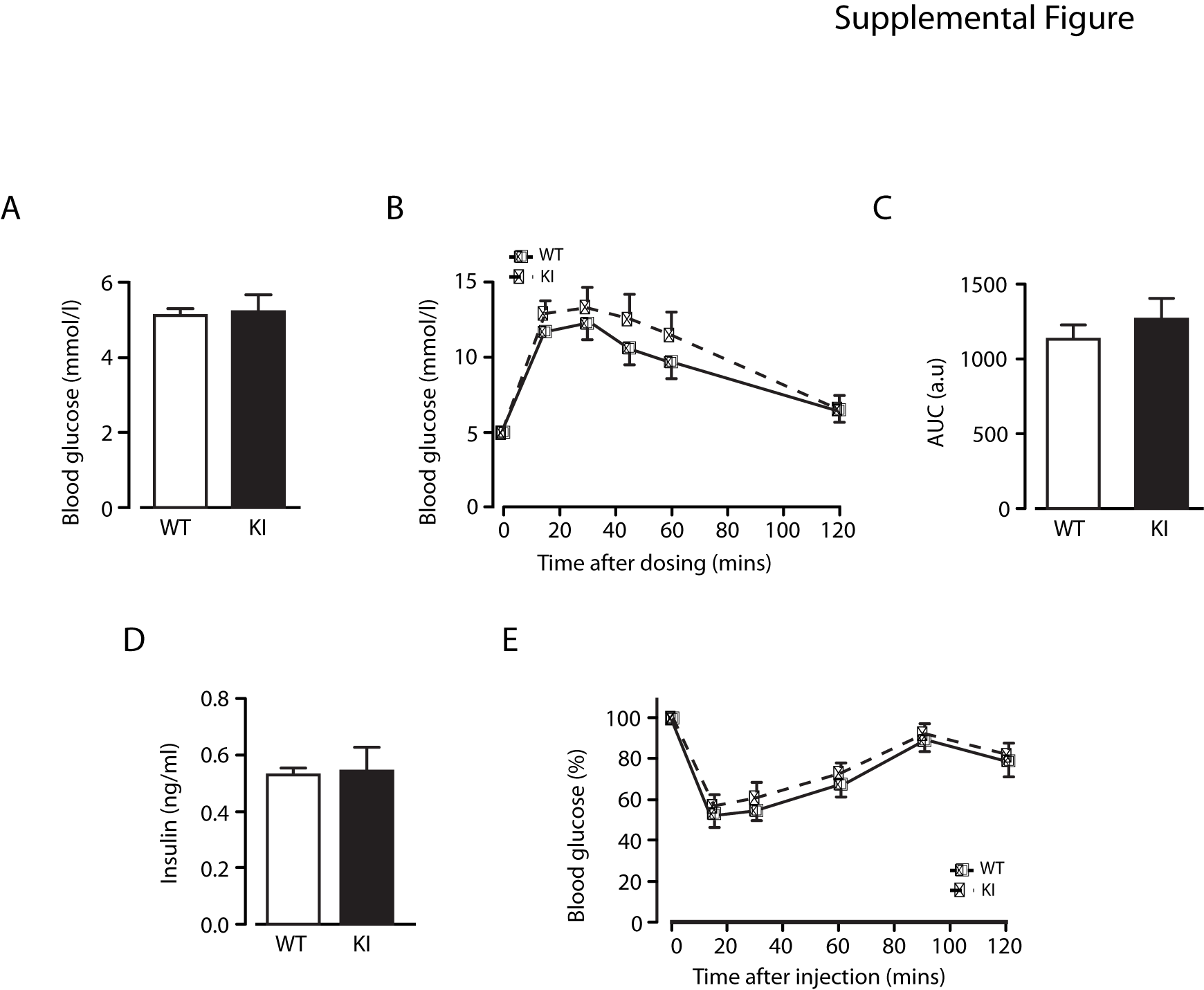

Supplement: S1 Fig — (A) Fasted blood glucose levels RC-fed mice of the indicted genotypes. (B) Intraperitoneal glucose tolerance tests were performed on RC-fed WT and KI mice, with quantification of the total glycaemic excursion (area under the curve; AUC) shown. (D) Fasted blood insulin levels for RC-fed WT and KI mice. (E) Insulin tolerance tests performed on RC-fed WT and KI mice. n = 4–8. (TIF) [file pone.0138872.s001.tif]
